# Performance Evaluation of Source Camera Attribution by Using Likelihood Ratio Methods

**DOI:** 10.3390/jimaging7070116

**Published:** 2021-07-15

**Authors:** Pasquale Ferrara, Rudolf Haraksim, Laurent Beslay

**Affiliations:** Joint Research Centre, European Commission, 21027 Ispra, Italy; rudolf.haraksim@ec.europa.eu (R.H.); laurent.beslay@ec.europa.eu (L.B.)

**Keywords:** forensic evidence evaluation, video source attribution, likelihood ratio, performance

## Abstract

Performance evaluation of source camera attribution methods typically stop at the level of analysis of hard to interpret similarity scores. Standard analytic tools include Detection Error Trade-off or Receiver Operating Characteristic curves, or other scalar performance metrics, such as Equal Error Rate or error rates at a specific decision threshold. However, the main drawback of similarity scores is their lack of probabilistic interpretation and thereby their lack of usability in forensic investigation, when assisting the trier of fact to make more sound and more informed decisions. The main objective of this work is to demonstrate a transition from the similarity scores to likelihood ratios in the scope of digital evidence evaluation, which not only have probabilistic meaning, but can be immediately incorporated into the forensic casework and combined with the rest of the case-related forensic. Likelihood ratios are calculated from the Photo Response Non-Uniformity source attribution similarity scores. The experiments conducted aim to compare different strategies applied to both digital images and videos, by considering their respective peculiarities. The results are presented in a format compatible with the guideline for validation of forensic likelihood ratio methods.

## 1. Introduction

Evaluation of forensic evidence relies on the concept of likelihood ratios (LRs), derived from the Bayes theorem. In fact, reporting LRs is the preferred way of presenting findings from criminal investigations across the spectrum of forensic disciplines [1]. This is reflected by a number of best-practice manuals [2,3] published by the European Network of Forensic Science Institutes (ENFSI)—covering disciplines of handwriting, fingerprints, document examination and others.

In the vast majority of cases, the result of a comparison between a questioned sample and the reference database leads to a similarity score, which is often dimensionless, lacking any kind of probabilistic interpretation and is therefore very difficult to incorporate into the forensic work-flow, unlike the LRs. It is the case of source camera attribution based on the Sensor Pattern Noise (SPN) or Photo Response Non-Uniformity (PRNU) [4,5], where most of the time the Peak to Correlation Energies (PCEs) [6] are compared to camera-related noise patterns.

Calculation of LRs from similarity scores is described in the literature [7,8,9,10,11,12,13,14,15], including a LR framework for camera source attribution using SPN and PRNU of still images [16]. Vast majority of these approaches use the plug-in scoring methods, which rely on post-processing of similarity scores using statistical modeling for computation of LRs. Direct methods, which output LR values instead of similarity scores have likewise been described in the literature [17]. These are much more complex to implement mainly due to the necessity to integrate-out the uncertainties when the feature vectors are compared under either of the propositions. The direct methods, as the title suggests, produce probabilistically sound LRs. Due to the continuous similarity score output of PRNU based methods, we use the plug-in score-based approach in order to facilitate a “fair” evaluation and inter-model comparison.

The main contribution of this article is the assignment of probabilistic interpretation to the set of similarity scores obtained from PRNU comparisons in the context of source camera attribution. This aim is reached by converting similarity scores into LRs within a Bayesian interpretation framework [18]. The performance of the resulting LR values, and by extension their usefulness for forensic investigation, is measured following the methodology developed in [19,20,21,22,23]. The objective is to reinforce the reliability of innovative tools such as source camera attribution, allowing them to be used not only as simple new investigation leads but also to contribute in a more determinant way to the investigation of digital forensic evidence. As underlined in the recently adopted EU strategy [24] to tackle Organized Crime 2021–2025, law enforcement and judiciary authorities need to fit for the digital age. The consolidation of their tactics and techniques for digital investigation with new approaches such as the one presented here, will reinforce the acceptability of those digital evidence submitted to the court.

The article is structured in the following way: in the next section we introduce the fundamentals of PRNU analysis. Section 3 presents the score-based plug-in Bayesian evidence evaluation methods for calculation of LRs and tools used for evaluation of performance of these methods. In Section 4, we describe the experimental protocol, the similarity scores and their mapping into LR values. Results obtained from a comparison of different methodologies are presented in Section 5. The contributions and future works are summarized in the conclusions in Section 6.

## 2. Prnu-Based Source Camera Attribution

PRNU is a unique noise pattern that every camera sensor implants like a passive watermark into every digital image [4,5] and video [25,26]. Due to its uniqueness, the extraction of PRNU signal allows to link a media content to its source device like a digital “fingerprint”. More in depth, PRNU is a 2D multiplicative noise pattern and can be modelled as a zero-mean white Gaussian noise [27], as a first approximation. Formally, a generic image can be described as

(1) $$I=I_{\left(0\right)}+I_{\left(0\right)}\cdot K_{I}+\Theta$$

where $I_{\left(0\right)}$ is an ideal noiseless image, $K_{I}$ is the PRNU and Θ is a noise term which considers other noisy contributions (i.e., dark current, quantization noise, etc.).

Several techniques were proposed to extract PRNU from an image but in this paper we refer to the one described in [28]. At image level, sensor noise is extracted by means of 2D discrete wavelet decomposition; then, saturated pixels are attenuated, and the noise pattern is normalized to erase liner patterns. Finally, ‘blockiness’ artifacts due to JPEG compression are removed by means of Wiener filtering.

As best practice, the PRNU associated to a given sensor is estimated by replicating the previous processing for a large enough set of flat-field images, in order to reduce the impact of the images content. The PRNU is then estimated according to the Maximum Likelihood criterion [28] as:

(2) $$\hat{K}\left(x,y\right)=\frac{\sum_{l}I_{l}\left(x,y\right)\cdot K_{l}\left(x,y\right)}{\sum_{l}I_{l}^{2}\left(x,y\right)}$$

where $I_{l}\left(x,y\right)$ and $K_{l}\left(x,y\right)$ are, respectively, the images and their associated PRNU estimate.

### 2.1. Peak-to-Correlation Energy

A similarity measure is needed in order to compare two PRNUs and classify whether they come from the same camera or not. Goljan et al. [6] proposed Peak-to-Correlation Energy (PCE) instead of correlation. PCE consists of measuring the ratio between the correlation peak energy and the energy of correlations evaluated for shifts outside from a neighborhood around the peak value. In order to calculate the PCE, the correlation matrix ϱ(u,v) between two noise pattern of size r×c needs to be computed in the following way:

(3) $$PCE=\frac{\varrho {\left(u_{0},v_{0}\right)}^{2}}{\frac{1}{rc-|N|}\sum_{(u,v)\notin \Omega }\varrho {\left(u,v\right)}^{2}}$$

where N is a neighborhood of |N| pixels surrounding the correlation peak in position $\left(u_{0},v_{0}\right)$.

### 2.2. Extension to Videos

**Figure 1:**
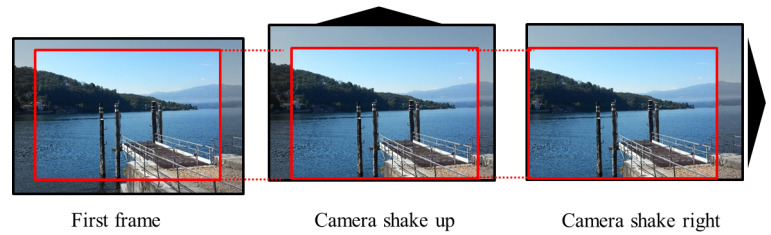
Digital motion video stabilization on subsequent frames. Undesired camera shakes are compensated for in order to have stable contents.

A straightforward solution for extracting a unique PRNU from a video is to consider video frames as images, and then to apply (2). This approach implicitly assumes geometric alignment of all noise patterns. Unfortunately, such an assumption does not hold for the most recent imaging devices which feature Digital Motion Stabilization (DMS). The DMS aims to generate high quality videos by minimizing any visual impact of vibrations and shaky hands which are often present when using hand-held devices, as illustrated in Figure 1. It performs a geometric alignment of each video frame according to the frame content. This processing alters the geometrical frame-by-frame alignment of the PRNU, so that the assumption of geometrical alignment between PRNUs of the frames is not true any more, consequently leading to worse PRNU estimates if (2) is applied.

In order to address DMS challenge, several matching strategies have been proposed in the literature [29,30,31,32,33]. Although authors propose different approaches, all are based on PCE as similarity measures.

### 2.3. Reference PRNU Creation

The objective of the analysis is to attribute or dissociate a questioned image or video to a specific device. As a first step, the reference PRNU needs to be extracted for the camera. In the case of the images, the process is quite straightforward: a set of flat-field images is acquired, from which the PRNU is extracted according to (2). In the case of the videos, the process is a bit more elaborated. There are at least two options proposed in the literature:

1. Using flat-field video recording to extract key-frame sensor noise and compute PRNU camera digital fingerprint according to (2). Still videos are used to limit the effect of motion stabilization. For the sake of simplicity, we name it RT1.
2. Employing both flat-field images and flat-field videos [34] in order to lessen the impact of motion stabilization as well as the impact of video compression, which is typically stronger for video frames compared to images. We name this second type RT2.

**Figure 2:**
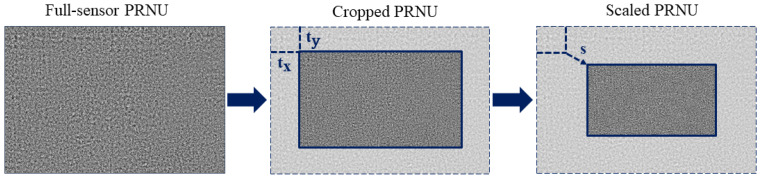
In-camera processing involved in video creation.

In order to use both, video recordings and images, we briefly recall how a camera generates a video frame. The process involves three steps: acquisition of a full-frame image, cropping of an internal region with a different aspect ratio (e.g., 16:9 for High Definition videos) and scaling to the final resolution. By assuming that the crop is symmetric with respect to the optical centre and posing the reference system at the centre of the frame, the relation between image and video PRNUs, namely $K_{I}$ and $K_{V}$, can be modeled by the scaling factor s∈R. Once the scaling factor is estimated [30,31,35], the PRNU extracted from images $K_{I}$ is resized accordingly, as shown in Figure 2.

### 2.4. Similarity Scores

As we explained in Section 2.1, similarity scores between two PRNU patterns are based on the computation of PCE. However, its computation does not follow a standard procedure, it is adapted from time to time to the particular use-case. For instance, if a certain robustness against image cropping is needed [6], the analyst can adopt an extended version of PCE where the position of the correlation peak is calculated as:

(4) $$\left(u_{0},v_{0}\right)\;=\;\underset{u,v\in U}{arg max}\left\{\varrho \left(u,v\right)\right\}$$

where *U* is an arbitrary neighbourhood in which the correlation peak is searched. The operation of maximization clearly impacts on the distribution of the similarity scores. Similar considerations can be made in the case of the video recordings, for those approaches that try to minimize the impact of DMS by adopting (4).

In order to simplify our analysis, we assume that no operation aiming to maliciously modify the PRNU is applied to the data. In this setting, the similarity scores for images are computed according to (3). In the case of the video recordings, we compared three different PRNU comparison strategies:

(a) Baseline: PRNU is obtained by cumulating the noise patterns extracted frame-by-frame according to (2), and the PCE is computed.
(b) Highest Frame Score (HFS): PRNU is extracted and compared frame-by-frame against the reference PRNU, and the maximum PCE is taken [30].
(c) Cumulated Sorted Frames Score (CSFS): PRNUs, extracted from each frame and compared with the reference signal, are first sorted in a descending order according to their individual PCE values; then, they are progressively cumulated, according to (2); finally, the maximum of PCE values obtained at each cumulation step is taken [31].

All the above-mentioned methods compute the PCE as described in (4).

Finally, it is worth noting that, according to the Equation (3), PCE can assume values in the range [0,+∞). Because in practice the PCE covers a very large range about 0 to ∼$10^{6}$, we consider a $\log_{10}$ scale.

## 3. Performance Evaluation

Couple of key components are necessary in order to compute LRs from the similarity scores: the ground truth regarding the source of origin of the image/video (same source or different source), a set of forensic propositions (hypotheses set for the defence and for the prosecution), and similarity scores, which are produced by different methods described in the previous section. Unlike the traditional performance assessment, which is usually limited to the analysis of the Detection Error Trade-off (DET) and Receiver Operating Characteristic (ROC) curves, we add the probabilistic meaning and interpretation to the similarity scores by transforming them into LRs. In order to do this, we set the hypotheses at the source level:

- $H_{P}$ (Prosecution): the Questioned Data (QD) comes from the camera C (mated trial).
- $H_{D}$ (Defense): the QD does not come from the camera C (non-mated trial).

It should be noted here that it is possible, and encouraged, to set the propositions at other than the source level [36]. Once the hypotheses are set, we proceed with the evaluation of forensic evidence under the Bayesian LR framework.

### 3.1. Bayesian Interpretation Framework

Different ways have been described in the forensic literature to calculate the LRs from continuous similarity scores [19,22]. Once the hypotheses are set, the strength of forensic evidence E is calculated in the following way:

(5) $$LR=\frac{P(E|H_{P},I)}{P(E|H_{D},I)}$$

where in the numerator of the *LR* we have the probability of observing E(QD) under the prosecution hypothesis (and additional related case information) and in the denominator of the *LR* we have the probability of observing the same evidence E(QD) under the defence hypothesis (and additional case-related information). We use a leave-one out cross-validation strategy [20], in which the role of evidence is taken by the left-out similarity score and the LRs are calculated in the following way:

(6) $$LR=\frac{f(S|H_{P})}{f(S|H_{D})}$$

where the f(·) represents the probability density function of the remaining scores and the *S* represents the left-out observation.

### 3.2. Performance Evaluation Tools

Performance assessment of the LR values under either of the propositions follows the methodology proposed in [19,21,22]. In their work on validation of LR values for forensic casework the authors propose measurement of two sets of performance characteristics—primary and secondary.

Given the limited amount of data we focus on evaluation of performance using the primary characteristics and leave the concept of validation of the LRs for forensic casework for future research. Although the full scope of the proposed “validation” framework cannot be applied, the basic concepts presented are valid and provide supplementary information, complementing the typically reported ROC/DET representations and accuracy measures at a fixed operating point.

The following performance characteristics and corresponding graphical representations are presented in the results section:

- *accuracy*, as sum of discriminating power and calibration, represented by the Empirical Cross Entropy (ECE) plot and measured by the log LR cost (CLLR) [37];
- *discriminating power* represented by the DET and ${\mathrm{ECE}}^{\min}$ plots and measured by the Equal Error Rate (EER) and ${\mathrm{CLLR}}^{\min}$ [38];
- *calibration* represented by the Tippet and the ECE plots and measured by ${\mathrm{CLLR}}^{\mathrm{cal}}$ [37].

## 4. Experimental Protocol

In this section, we first describe the data set we used in the experiments. Afterwards, the experimental protocol follows a logical separation, based on the type of data, namely images and video recordings. For videos, we separate the analysis in function of the type of PRNU reference and the presence or the absence of DMS, in order to perform the four basic experiments mentioned in Section 5. All the experiments produce a set of similarity scores calculated in the course of comparison between the questioned and reference samples.

### 4.1. Data Corpus

We used the Vision Dataset [39] (except for device D13, according to the names convention) to create a benchmark dataset for pictures and videos. Among the devices, 16 produce motion stabilized videos, whereas the other 18 produce only non-stabilized videos. For each device, we have at our disposal:

- A set of 30 randomly selected flat-field images, from which we extracted the image PRNU $K_{I}$.
- A set of flat-field static (labelled as still) and moving (labeled as panrot and move) videos. These videos are used to create reference PRNU $K_{V}$ per device.
- A set of images with natural content that we used as query data. The set is composed of at least 200 pictures per devices.
- A set of non-flat query videos including still, pan-rotating and moving videos.

In summary, we used 34 different devices, 34 × 30 = 1020 flat-field images, 218 flat-field video recordings, 7393 natural images, 223 non-stabilized and 190 stabilized questioned videos. The number of mated and non-mated scores is summarized in Table 1.

### 4.2. Preliminary Analysis of the Similarity Scores

**Figure 3:**
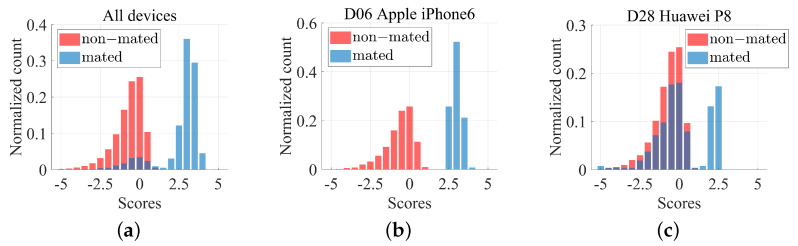
Histograms of empirical score distributions obtained from images. (**a**): empirical distributions by considering all the devices within the benchmark dataset. (**b**): scores obtained from query images coming from an Apple iPhone 6. (**c**): scores distributions for images acquired through a Huawei P8.

The two types of experiments (images and videos) present slightly different challenges. For example, let us consider the scores distributions obtained from images analysis and shown in Figure 3. The empirical distributions of $P(E|H_{P},I)$ and $P(E|H_{D},I)$ are overlapping to some extent. At the same time, if we look closer at the distribution for each device, we observe that for some devices, see Figure 3b, the two distributions are perfectly separated. On the other hand, for some other devices, the score distributions show a non-negligible proportion of mated similarity scores attaining the non-mated similarity score magnitudes, effectively heavily contributing to the False Rejection rates Figure 3c. In other words, the PRNU obtained from these devices compromises the overall performance of the methods under evaluation.

**Figure 4:**
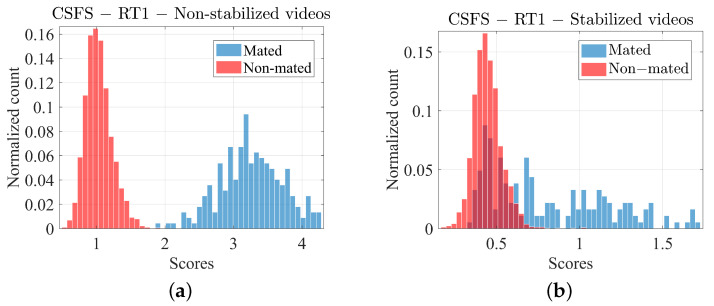
Histograms of empirical score distributions obtained from non-stabilized (**a**) and stabilized video recordings (**b**). The scores are obtained by using the reference Photo Response Non-Uniformity (PRNU) of type RT1 and by applying the Cumulated Sorted Frame Score (CSFS) method.

In some cases, for example non-stabilized video recordings against RT1 result in “perfect separation” of the mated and non-mated score distributions (see Figure 4a). While the perfect separation is highly desirable, in the case when number of comparisons is relatively small (as in our case), it usually points in the direction of one of the following problems (or a combination of any of these): plain lack of data, over-fitting (sub-optimal separation of the dataset into training and testing subsets), or a feature space being much greater than the actual dataset.

For some cases however, such as PRNUs obtained from the images, a significant proportion of the mated scores attains the magnitudes of the non-mated scores, thus contributing heavily to the False Rejection error rates. Again, PRNU obtained from these devices compromises the overall performance of the methods under evaluation.

### 4.3. Score to LR Calibration Transformation

#### 4.3.1. Images

Our analysis into the distribution of similarity scores produced by the image test and reference samples showed that the mated samples ($H_{P}$) were distributed following the inverted chi squared probability distribution function (PDF) with 1 degree of freedom and shape parameter equal to zero [28]. The non-mated similarity scores ($H_{D}$) followed a similar PDF with 1 degree of freedom and a non-zero shape parameter.

Although the inverted chi-squared PDF’s provided a reasonably good estimate, they did not generalize well to the previously unseen data when subjected to cross-validation. The generalization issue, or in our case inability to generalize well to the previously unseen data, can be explained by large inter and intra variability among the sensors embedded within different devices, even when coming from the same manufacturer.

Since we do not have at our disposal a fully exhaustive database of mobile devices/cameras from different manufacturers, we opted for a simpler solution and transformed the similarity scores into LRs using regularized logistic regression with a uniform prior regularization [40]. The process of calibration using linear logistic regression can be described in the following way:

- Iterative use of leave-one-out cross validation for both mated and non-mated scores, where each of the left-out scores “plays” the role of the evidence;
- One-to-one mapping from probability to log-odds domain is performed using a logit function [37];
- Calibrated LRs are calculated iteratively for each evidence score.

More detailed description of the use of LR calibration is beyond the scope of this article, but the reader might refer to [23] for more details.

#### 4.3.2. Video Recordings

In the case of the video recordings, we note that while the similarity scores under the hypothesis $H_{P}$ for the non-stabilized videos follows a Gaussian-like distribution in the logarithmic scale (see Figure 4a), the analogous similarity scores for stabilized videos do not (see Figure 4b). We therefore adopt a different calibration strategy for both cases.

Score distributions under hypothesis $H_{D}$ follow in both cases a Gaussian-like distribution in the logarithmic domain. This result is in agreement with the outcome of [31], where authors demonstrated that scores under hypothesis $H_{D}$ are distributed according to a Generalized Extreme Value [41] distribution on the linear scale.

The fact that both mated and non-mated distributions are positive indicates the need of calibration. In the subsequent step we perform a leave-one-out cross-validation calibration and calculate the LR values at the same time.

Knowing the ground truth regarding the origin of the pair of videos and the reference sample (RT1 or RT2), we proceed iteratively through the set of similarity scores, exclude one similarity score (mated or non-mated) to “play” the role of observed evidence. We use remaining similarity scores to model score distributions under either of the propositions.

The Gaussian calibration with optimal risk smoothing is used for the non-stabilized videos as both, the mated ($H_{P}$) and non-mated ($H_{D}$) scores resemble a “well-behaved” normal distribution (Figure 4a).

The calibration for the case of non-stabilized video sequences can be summarized in the following steps:

- Iterative use of leave-one-out cross-validation for mated and non-mated scores, where each of the left-out scores “plays” the role of the evidence;
- A normal distribution is fitted to the rest of the mated and non-mated scores;
- Calculation of the numerator and denominator of the LR for each left-out score;
- Calibrated LRs are calculated according to (6).

More detailed description of the calculation of LR values from normally distributed similarity scores is beyond the scope of this article, but the interested reader is kindly referred to [20] for more details.

Similarity scores, in particular the mated scores ($H_{P}$) produced in the course of comparison between the stabilized videos and reference PRNU do not follow any obvious distribution pattern (Figure 4b). In fact, it is very difficult to fit any particular distribution, given the fact that the mated comparison counts drop to zero on multiple occasions. One could argue that a kernel density function could serve the purpose with which we in principle agree, however given the relatively small number of comparisons we opted for a linear logistic regression calibration in a process identical to that described above in Section 4.3.

## 5. Performance Evaluation Results

In this section, we provide the experimental results of the PRNU source attribution presented in the likelihood ratios framework. Alike the experimental protocol Section 4, results section follows the same comparative analysis between images, stabilized and non-stabilized videos.

### 5.1. Images

By assuming that the images are exactly like the ones that the device produces, the most significant parameter that affects the PRNU is the image resolution, which varies from one camera model to another. For this reason, we repeated our analysis for three different resolutions: 1024×1024, 512×512 and 256×256, in order to see the effects of the resolution on the performance of the PRNU.

**Figure 5:**
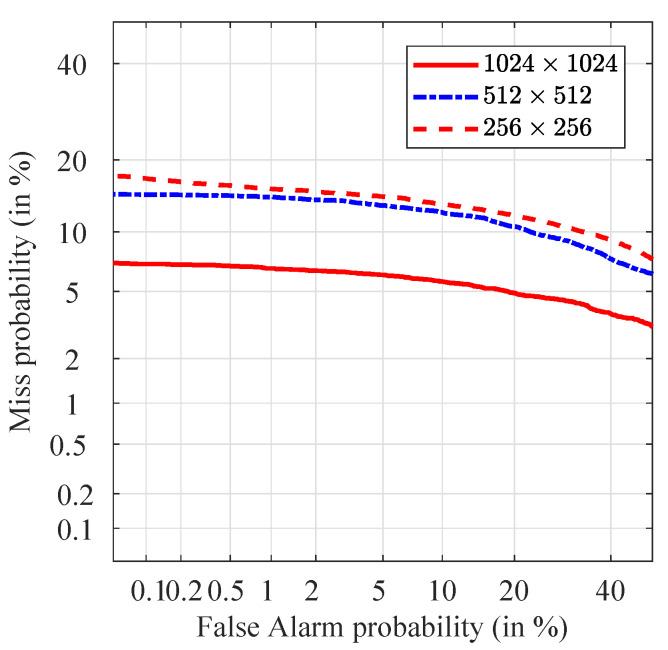
Detection Error Trade-off (DET) plots for picture at different resolutions: 1024×1024, 512×512 and 256×256.

The DET plots present the discriminating capabilities of the different methods. They (Figure 5) show the probability of false acceptance versus the probability of false rejection of the non-stabilized video on a Gaussian-warped scale. The main advantage of this representation over ROC curves is that the DET curves get close to linear when the LR values follow Gaussian distribution. At the intersection of each DET curve with the main diagonal we find the EER which is a measure of discrimination [37]. The best discriminating capabilities were observed for the highest tested resolution (1024×1024) with the ERR 6%. Reducing the image resolution to one fourth (512×512 pixels) significantly reduce the discriminating capabilities of the PRNU and nearly doubles the EER = 11.8%. Additional reduction of the image size to 256×256 pixels lower the discriminating capabilities and rises the EER to 12.8%.

**Figure 6:**
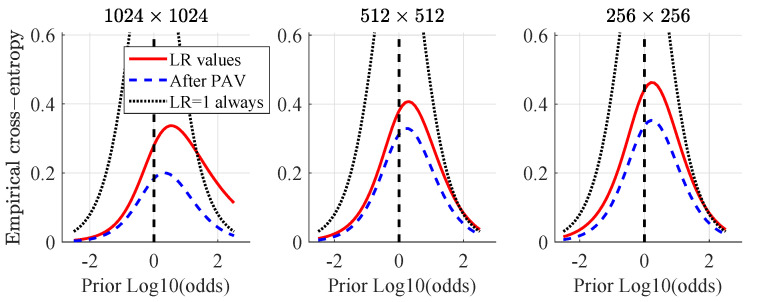
Empirical Cross Entropy (ECE) plots for pictures at different resolutions: 1024×1024, 512×512 and 256×256.

Figure 6 shows the Empirical Cross-Entropy plots, which have information-theoretical interpretation [42]. They provide summary of accuracy, discriminating capabilities and a calibration of a given method, conveniently all in one plot. The black dotted line represents a neutral system (effectively equivalent to making decisions based on a coin-toss using a fair coin). The red line shows the measure of accuracy (CLLR) at the prior-$\log_{10}$-odds = 0, blue dashed line shows the measure of discriminating capabilities of a method (${\mathrm{CLLR}}^{\mathrm{MIN}}$) at the prior-$\log_{10}$-odds = 0. The difference between the CLLR and ${\mathrm{CLLR}}^{\mathrm{MIN}}$ is a measure of calibration (${\mathrm{CLLR}}^{\mathrm{CAL}}$). When the LRs support the correct hypotheses, the CLLR values tend to be lower (e.g., the lower the CLLR the better the accuracy, the lower the ${\mathrm{CLLR}}^{\mathrm{MIN}}$ the better the discriminating capabilities and the lower the ${\mathrm{CLLR}}^{\mathrm{CAL}}$ the better he calibration of a given method).

As already introduced in the DET plots, the best discriminating capability of the PRNU is observed for 1024×1024 images, confirmed in the ECE plots, achieving ${\mathrm{CLLR}}^{\mathrm{MIN}}$ of 0.18. It also shows the highest overall accuracy out of the three image resolutions considered with CLLR = 0.28. Although showing the best discriminating capabilities and accuracy, this method presents the second worst calibration with the calibration loss equal to one third of the overall accuracy (${\mathrm{CLLR}}^{\mathrm{CAL}}$ = 0.096). ECE curves, unlike the DET plots, reveal a weak spot. At prior-log-odds = 1.8 the CLLR (red curve of the 1024×1024 images) crosses the line of the reference system (black dotted line), effectively making decisions at the prior-log-odds >1.8 worse than a coin toss using a fair coin.

**Figure 7:**
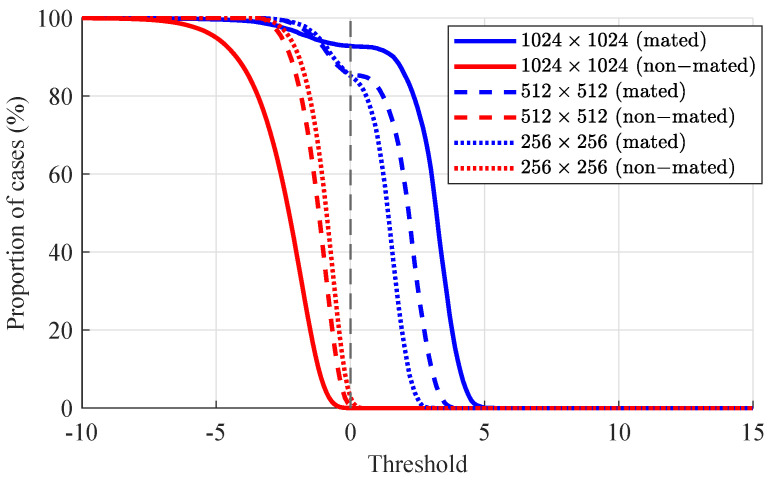
Tippet plots for pictures at different resolutions: 1024×1024, 512×512 and 256×256. Cumulated distributions of mated (blue) and non-mated (red) scores are presented.

Tippett plots as additional measure of calibration presented in Figure 7 show cumulative distribution functions of LRs [38]. Individual curves represent the proportion of comparisons supporting either of the two propositions. The rates of misleading evidence are observed at the intersection of the Tippett plots with the $\log_{10}\left(\mathrm{LR}\right)\;=\;0$. The symmetry between the two curves (supporting either of the propositions) is likewise used as an indicator of calibration.

The the lowest probabilities of misleading evidence are observed for 1024×1024 resolution images (${\mathrm{PME}}_{HP}=7.074\%$ and ${\mathrm{PME}}_{HD}=0.02\%$), and complement the calibration results indicated by the ECE plots above. The probabilities of misleading evidence for the 512×512 and 256×256 resolution images are show in Table 2.

### 5.2. Non-Stabilized Video Recordings

**Figure 8:**
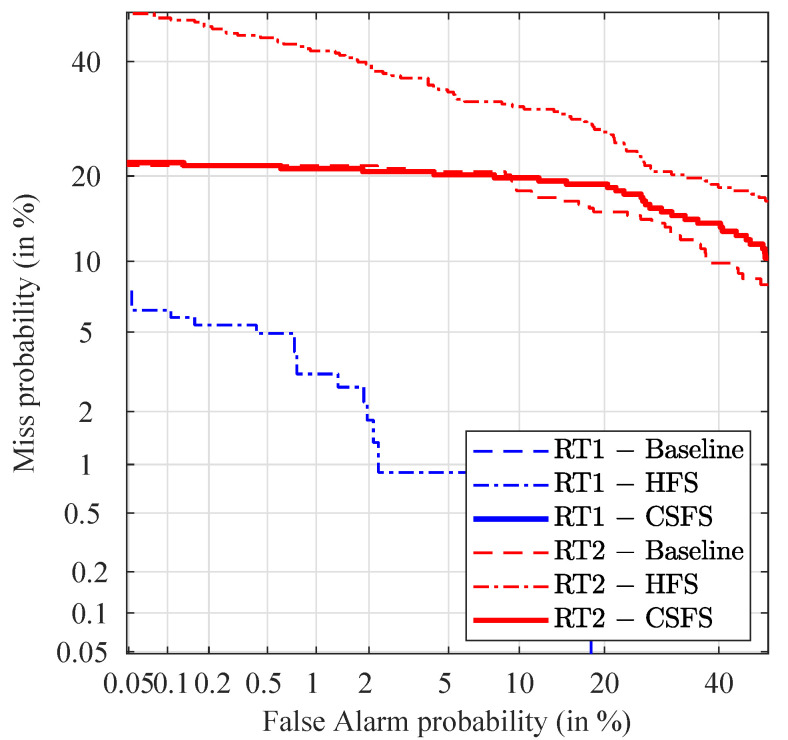
DET plots for non-stabilized videos.

DET curves in the case of non-stabilized videos are shown in Figure 8. As an element of comparison, it should be noted here that the discriminating capabilities of well-established biometric systems produce EER typically below 5%, which is also true for some of the methods presented in the non-stabilized subsection. The relatively high EER values achieved with the stabilized video recordings, in contrast with the non-stabilized videos point out potential for additional improvement.

The baseline method shows the best discriminating capabilities in terms of EER in case of comparison of non-stabilized videos against the reference for both types of reference PRNU. The proposed method offers identical or comparable performance (in the worst case, 1% of loss). Due to the near-perfect separation of the mated and non-mated scores, the baseline method and the CSFS method are not visible in the DET plot as their EERs are close to zero.

**Figure 9:**
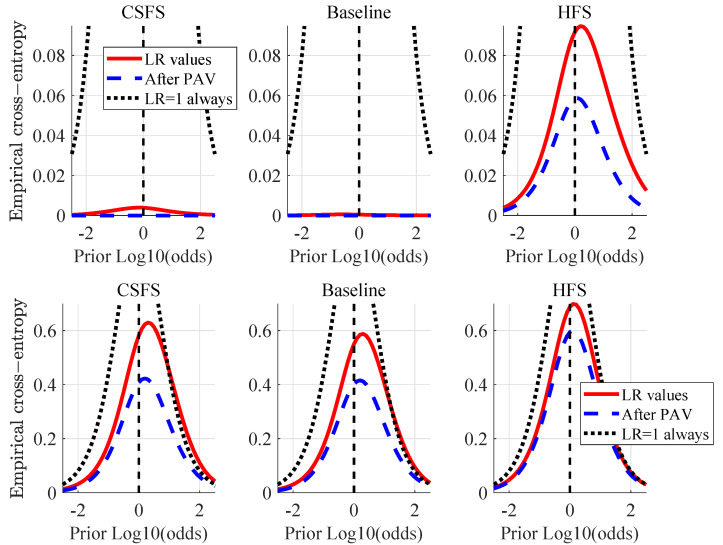
ECE plots: non-stabilized videos vs. RT1 (first row) and RT2 (second row).

Among the methods compared by means of ECE plots (Figure 9), the baseline method shows the best performance in terms of discriminating capabilities and accuracy for the comparisons of non-stabilized videos versus RT2. The best accuracy and discriminating capabilities in the case of comparisons against reference RT1 is nearly identical for the CSFS and the baseline method, while the baseline method shows slightly better calibration. It is worth adding that the differences observed between these two methods are negligible. Accuracy of LR values produced by the CSFS and the baseline method show sub-optimal performance for the prior-$\log_{10}$-odds ≥ 1, where the red line crosses the black dotted line. LRs of both of these methods in this region are unreliable [20] and the fact-finder trusting these will be effectively making worse decisions than using a coin-toss. Further tests using different calibration methods are necessary to eradicate the source of this behaviour.

**Figure 10:**
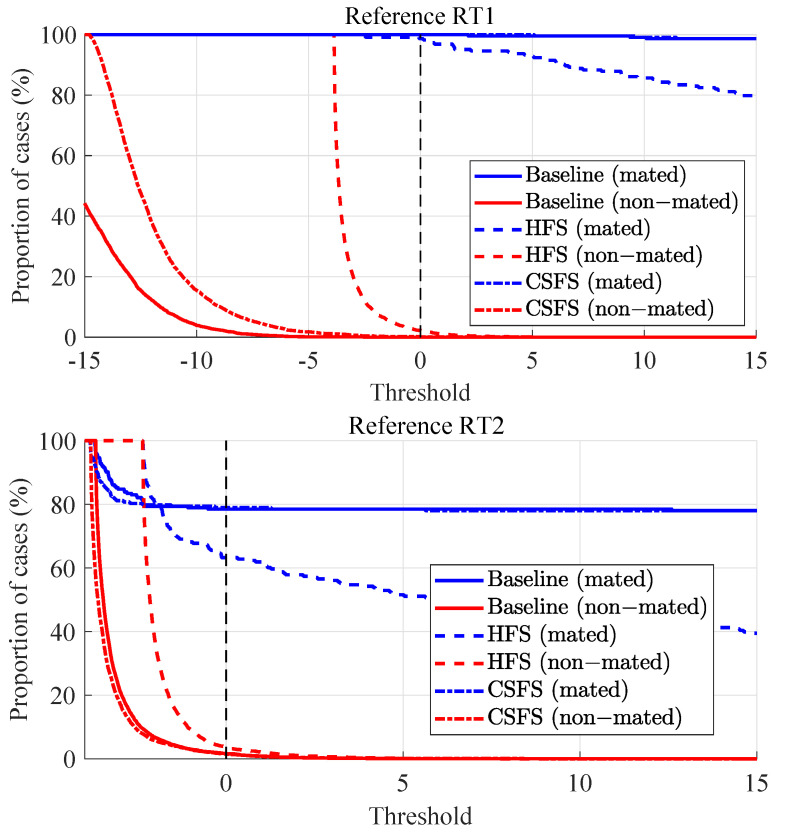
Tippett plots: non-stabilized videos vs. RT1 (upper) and RT2 (lower).

By looking at the Tippett plots (Figure 10), the lowest probabilities of misleading evidence in the case of non-stabilized videos in the scope of RT2 experiments is observed for the CSFS method. On the other hand, lowest probability of misleading evidence in the case of non-stabilized videos in the scope of RT1 experiments supporting the $H_{P}$ is observed for the CSFS method and supporting the $H_{D}$ for the baseline method. It should likewise be noted that on average, lower rates of misleading evidence have been observed in the context of RT1 experiments, which means that LR in this case provide stronger support to the correct propositions. The results for the non-stabilized videos are summarized below in Table 3 (the best performance is highlighted in bold).

LRs produced in the course of non-stabilized videos show “perfect” accuracy and calibration when compared in the scope of RT1 experiments for proposed and baseline methods. Given the relatively small dataset, these results should be further analysed and followed up by a series of experiments to show the robustness of methods to the previously unseen data and potential overfitting. Slightly better accuracy and calibration was observed for the baseline method when comparing RT2 video recordings however, lower rates of misleading evidence were observed for the proposed method. In general, the performance of baseline and proposed methods can be considered equivalent. Decisions based on the LR values observed for prior log10odds greater than 1.0 for the questioned videos in the scope of RT2 experiments should not be trusted due to the fact that the ECE curve crosses the reference line and these decisions are effectively worse than decisions based on a coin toss.

### 5.3. Stabilized Videos

**Figure 11:**
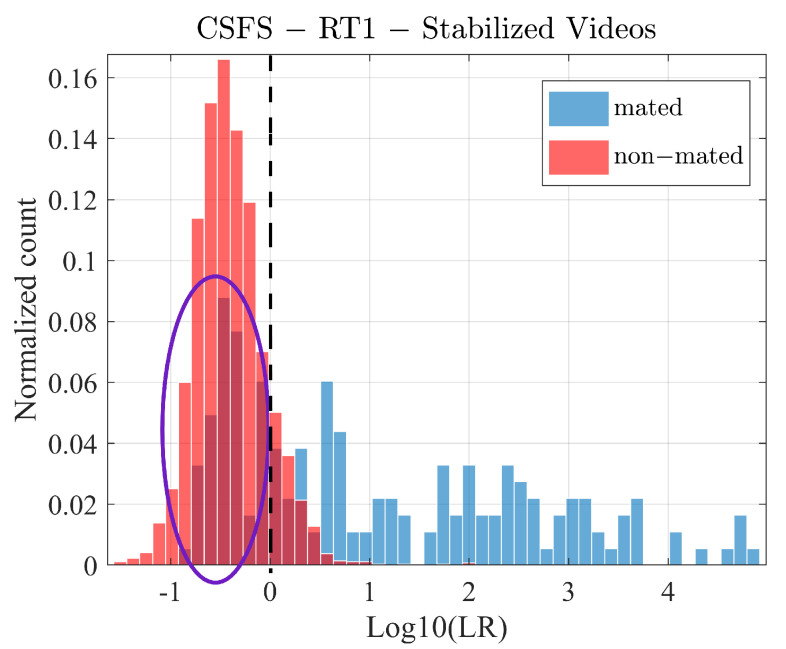
Likelihood ratio distribution after the linear logistic regression calibration. Magenta ellipse indicates the issue with the mated scores, black line shows $log_{10}\left(LR\right)=0$.

**Figure 12:**
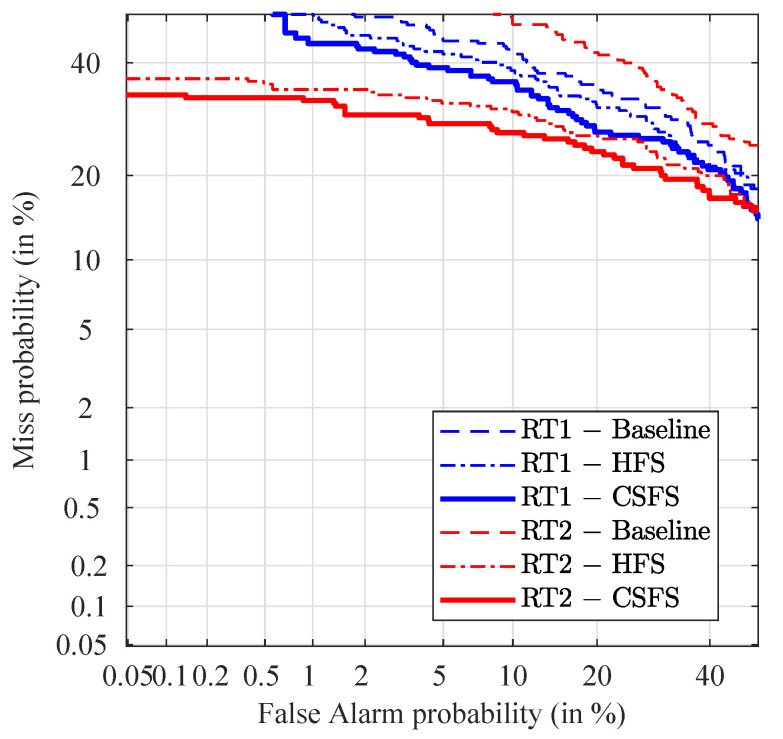
DET plots for stabilized videos.

Before discussing the results, we provide an analysis of the resulting LR values for the stabilized videos by means of normalized-count histograms, which perfectly suit the purpose. As shown in Figure 11, a significant proportion of the LRs supporting the $H_{P}$ proposition (blue histogram) is overlapping with the LRs supporting the $H_{D}$ proposition (red histogram). As a result, all of these LRs provide support to the wrong hypothesis ($H_{D}$). From the two groups of the stabilized videos (compared against the reference RT1 or RT2) we conclude that the method showing the best discriminating capabilities is in both cases the CSFS method (see Figure 12). The CSFS method shows the best performance in terms of EER for comparisons of stabilized images against the reference set of both types of reference PRNU.

**Figure 13:**
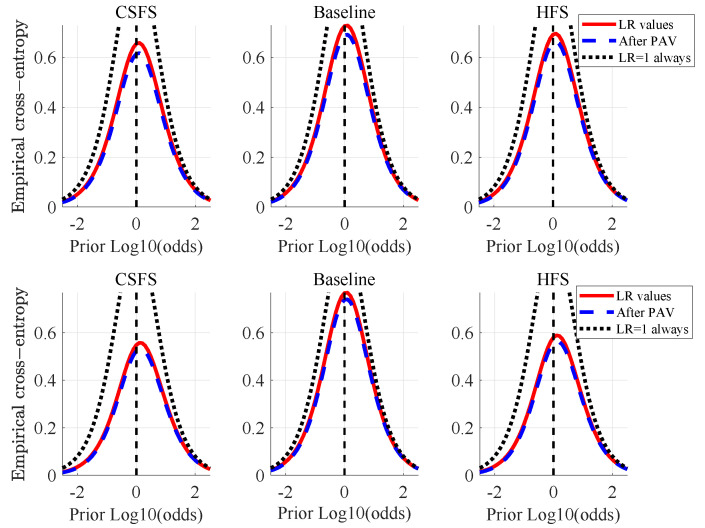
ECE plots: stabilized videos vs. RT1 (first row) and RT2 (second row).

Figure 13 shows the ECE plots in the case of stabilized videos. Amongst the methods compared, the CSFS method shows the best performance in terms of discriminating capabilities and accuracy, while the HFS method shows the best calibration (all be it the difference in calibration between the method proposed and the HFS method is negligible and both of these methods can be described as rather well calibrated).

**Figure 14:**
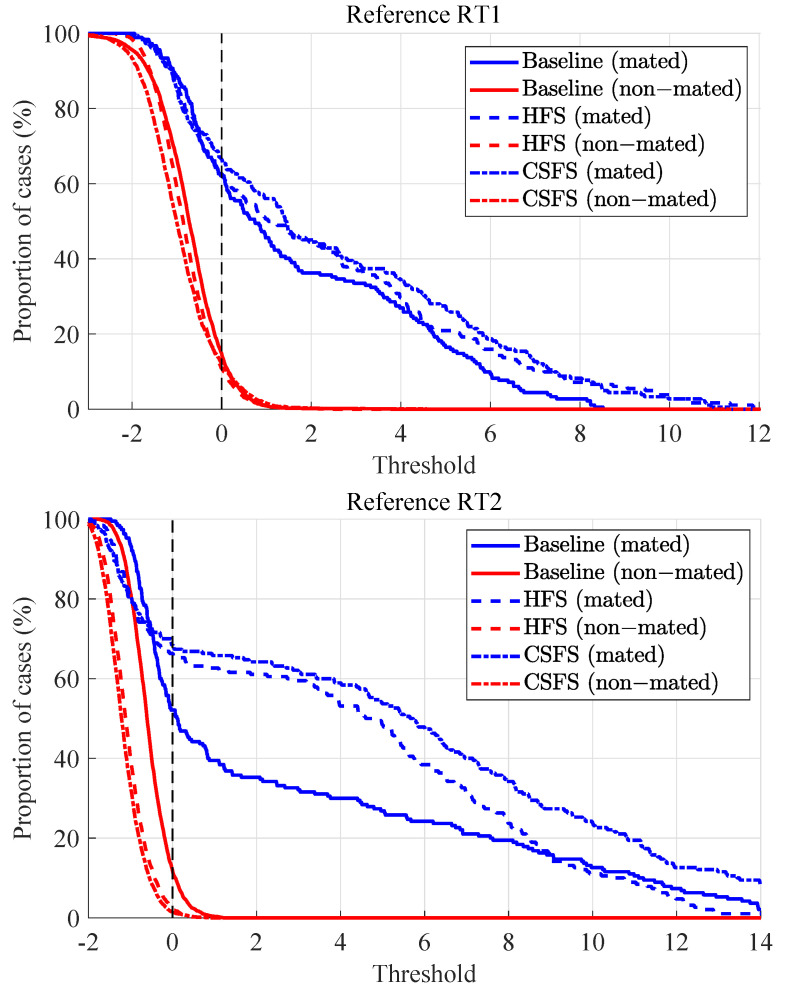
Tippett plots: stabilized videos vs. RT1 (upper) and RT2 (lower).

High rates of misleading evidence of the LR’s supporting the $H_{P}$ on average are the result of small similarity scores (which resulted in low LR values) observed for mated comparisons as discussed above (see Figure 14).

The results for the stabilized video recordings compared against reference RT1 and RT2 are summarized in Table 4 (the best performance is indicated in bold). LRs produced during stabilized videos experiments show better performance in terms of accuracy and discriminating power for the CSFS method over the remaining two methods. In the case of videos compared against RT1 reference the best calibration was observed for the HFS method. It should be noted that the calibration losses observed in the course of this set of experiments were minimal and decisions regarding which method to favour should not be based on the calibration measure alone.

## 6. Conclusions

In this article we addressed to our best knowledge for the first time the challenge of source camera attribution for video recordings from a perspective of a forensic evidence evaluation using likelihood ratios, and complemented previous research [16] on source camera attribution for still images. We have taken multiple continuous sets of similarity scores (mated and non-mated), converted them into LRs using the probability density function and measured their performance. In essence, we gave the difficult-to-interpret set of similarity scores a probabilistic meaning and interpretation.

Reflecting on the analysis of the results of different methods and settings, particularly ECE plots prove useful as they point out regions where produced LRs provide unreliable support to forensic evidence for both still images as well as video recordings. Considering the fact that there is a lot more information present in the video recordings (sequence of images) than in a single still image, it is not surprising that the best performance in terms of accuracy, calibration and discriminating capabilities was observed for the non-stabilized video recordings. However, performance dramatically drops if digital motion stabilization is adopted. A particular attention should be paid to the analysis of images, for which apart from the image resolution the device model should be considered as a deciding parameter. The latter might affect in a positive or negative manner the overall performance of the system.

Additional validation experiments accompanied by further analysis of the similarity scores will be performed in the near future. Particular attention will be given to the “perfectly separated” similarity scores and regions of high correlation, with the aim to demonstrate robustness to the lack of data, generalization and coherence [23]—which present the secondary performance characteristics necessary for the validation of the methods presented for forensic casework. Likewise, different probability distribution functions will be used to convert the hard-to-interpret similarity scores into reliable likelihood ratios.

## Figures and Tables

**Table 1:** Number of similarity scores per experiment.

	# Mated Scores	# Non-Mated Scores
Images	7393	243,969
Non-stabilized videos	223	3791
Stabilized videos	190	2850

**Table 2:** Performance metrics observed for different resolutions of the images. The best performance is highlighted in bold.

	Image Resolution		
	1024×1024	512×512	256×256
(%) EER	**5.984**	11.83	12.83
CLLR	**0.2798**	0.3802	0.4428
${\mathrm{CLLR}}^{\mathrm{MIN}}$	**0.1836**	0.3127	0.3377
${\mathrm{CLLR}}^{\mathrm{CAL}}$	0.09614	**0.06744**	0.1051
$\left(\%\right){\mathrm{PME}}_{HP}$	**7.074**	14.12	14.27
$\left(\%\right){\mathrm{PME}}_{HD}$	**0.2049**	1.347	5.24

**Table 3:** Summary of the results for accuracy, discriminating power and calibration for the non-stabilized videos. The best performance is highlighted in bold.

	RT1			RT2		
	CSFS	Baseline	HFS	CSFS	Baseline	HFS
(%) EER	**0.08**	**0.08**	1.98	17.43	**16.48**	23.85
CLLR	0.004	**0.003**	0.092	0.58	**0.55**	0.69
${\mathrm{CLLR}}^{\mathrm{MIN}}$	**0.003**	**0.003**	0.062	0.41	**0.4**	0.59
${\mathrm{CLLR}}^{\mathrm{CAL}}$	0.001	**0**	0.03	0.17	**0.15**	0.1
$\left(\%\right)\;{\mathrm{PME}}_{HP}$	**0**	**0**	1.34	**21.07**	21.5	36.77
$\left(\%\right)\;{\mathrm{PME}}_{HD}$	0.13	**0**	2.24	**1.5**	1.66	3.66

**Table 4:** Summary of the results for accuracy, discriminating power and calibration for the stabilized videos. The best performance is highlighted in bold.

	RT1			RT2		
	CSFS	Baseline	HFS	CSFS	Baseline	HFS
(%) EER	**26.46**	30.85	28.64	**22.7**	33.5	25.86
CLLR	**0.66**	0.73	0.69	**0.55**	0.77	0.58
${\mathrm{CLLR}}^{\mathrm{MIN}}$	**0.62**	0.69	0.66	**0.52**	0.74	0.56
${\mathrm{CLLR}}^{\mathrm{CAL}}$	0.04	0.04	**0.03**	**0.03**	**0.03**	0.04
$\left(\%\right){\mathrm{PME}}_{HP}$	**33.52**	37.91	37.36	**31.58**	47.89	33.68
$\left(\%\right){\mathrm{PME}}_{HD}$	12.37	15.07	**10.9**	**1.47**	12.35	2.49

## Data Availability

Publicly available datasets were analyzed in this study. This data can be found here: https://lesc.dinfo.unifi.it/VISION/ (last accessed: 23 September 2020).

## Abbreviations

The following abbreviations are used in this manuscript:

PRNU	Photo Response Non-Uniformity
DMS	Digital Motion Stabilization
LR	Likelihood Ratio
ENFSI	European Network of Forensic Science Institutes
SPN	Sensor Pattern Noise
PCE	Peak-to-Correlation Energy
RT1	Reference Type 1
RT2	Reference Type 2
HFS	Highest Frame Score
CSFS	Cumulated Sorted Frame Score
QD	Questioned Data
ROC	Receiver Operating Characteristic
DET	Detection Error Trade-off
ECE	Empirical Cross Entropy
CLLR	Curves and Log LR (cost)
EER	Equal Error Rate
